# Gastroduodenal Intussusception After Conversion Of Gastric Plication To Roux-En-Y Gastric Bypass: A Case Report With Intraoperative Video

**DOI:** 10.1007/s11695-025-07883-9

**Published:** 2025-04-23

**Authors:** Asyadée Jacob, Uto Randone, Ibrahim Dagher, Hadrien Tranchart

**Affiliations:** 1 Antoine-Béclère Hospital, Clamart, France; 2 University of Paris-Saclay, Gif-Sur-Yvette, France

**Keywords:** Intususception, Gastric plication, Roux-en-y gastric bypass

Bariatric surgery is a highly effective long-term treatment for obesity, leading to sustained weight loss. However, it is not without complications. One recognized postoperative issue following Roux-en-Y gastric bypass for morbid obesity is intestinal obstruction [1]. Various causes have been identified, including volvulus, adhesions, internal hernias, incarcerated ventral hernias and intussusception.

Intussusception, though rare, is a serious and potentially life-threatening long-term complication whose etiology remains largely unclear. The most common form (70%) involves retrograde intussusception of the common channel towards the jejunojejunostomy [1]. In contrast, anterograde intussusception of the remnant stomach into the duodenum is an exceptionally rare occurrence, with only a few cases reported in the literature [2–4].

Herein, we present a case of anterograde gastroduodenal intussusception after Roux-en-Y gastric bypass.

A 45-year-old female patient was referred to our emergency department for evaluation of postprandial abdominal pain persisting for two months, which had recently worsened and was accompanied by nausea.

Her medical history included a laparoscopic gastric plication performed four years ago for class II obesity (BMI: 37.6 kg/m^2^), which was later converted to a laparoscopic Roux-en-Y gastric bypass one year ago due to inadequate weight loss. The remnant stomach was preserved. This procedure resulted in a successful total weight loss of 30%.

On clinical examination, the patient presented with generalized abdominal distension and a tender mesogastric mass, though no signs of peritoneal irritation were observed. Laboratory findings revealed an elevated white blood cell count and C-reactive protein (CRP) levels, along with increased transaminases and hyperlipasemia.

A CT-scan was performed and revealed remnant stomach anterograde intussusception signs with presence of « target sign» (Fig. 1).

**Fig. 1:**
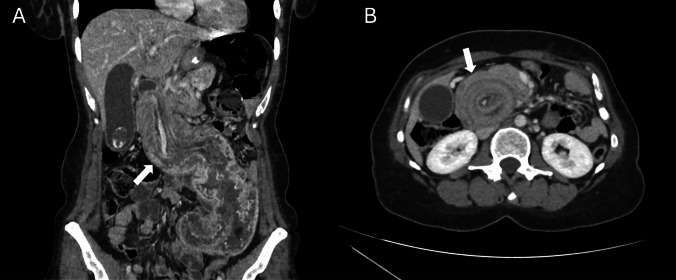
Frontal view of abdominal computed tomography scan revealing remnant stomach intussusception through the duodenum (white arrow, **A**). Axial view of abdominal computed tomography scan showing a « target sign» (white arrow, **B**)

An immediate laparoscopic exploration was attempted but required conversion to laparotomy. A gentle and gradual reduction of the remnant stomach, along with the gastroepiploic and left gastric vessels, was successfully performed. To prevent recurrence, resection of the remnant stomach was undertaken (Video 1).

Histopathological analysis of the resected stomach confirmed a tumor-free specimen with no signs of gastritis or ulcerations. The patient experienced an uneventful recovery and was discharged ten days postoperatively.

We report a rare case of anterograde gastroduodenal intussusception following Roux-En-Y gastric bypass. To our knowledge, only three other cases have been reported in the literature [2–4], all occurring in the context of revisional RYGBP. In all of these cases, the laxity of the remnant stomach was increased by initial gastro-splenic vessels division: during gastric plication in three cases including ours or by gastric division under the level of the gastrosplenic ligament in a patient that had prior biliopancreatic diversion by Scopinaro.

Gastric greater curvature plication following gastrosplenic vessel divisions may itself be a procedure that predisposes patients to gastroduodenal intussusception. Freitas et all. reported two patients who developed intussusception several months after gastric plication [5]. The authors hypothesized that intussusception may be an underreported complication of this procedure. Generally, gastrointestinal intussusceptions are secondary to intraluminal masses [5]. Gastric plication, by definition, creates an endoluminal mass, which, when combined with gastric release, could favor this complication.

In the present case, gastric plication likely created a lead point that predisposed the stomach to intussusception following its division during the Roux-en-Y Bypass surgery. As with isolated gastric plication, this complication may be underestimated. Indeed, our patient experienced two months of persistent abdominal pain before diagnosis, and another reported case presented only with chronic anemia. Consequently, systematic preventive resection of the remnant stomach or, at least, surgical gastropexy should be considered when performing revisional Roux-en-Y gastric bypass after gastric plication.

Gastroduodenal intususception following revisionnal Roux-en-Y Gastric Bypass is an exceptionally rare but potentially underdiagnosed event. Prior gastric plication appears to be a common predisposing factor for this complication. Surgeons should be aware of this phenomenon and preventive measures such as remnant gastric resection or gastropexy should be discussed when converting to Roux-en-Y Gastric Bypass.

## Supplementary Information

Below is the link to the electronic supplementary material.

Supplementary file1 (MP4 195 MB)

## Data Availability

No datasets were generated or analysed during the current study.

## References

[1] Singla S, Guenthart BA, May L, et al. Intussusception after laparoscopic gastric bypass surgery: An underrecognized complication. Minim Invasive Surg. 2012;2012:464853. 10.1155/2012/464853. 22991661 10.1155/2012/464853 PMC3444049

[2] Hillenbrand A, Waidner U, Henne-Bruns D, et al. After 3 years of starvation: Duodenum swallowed remaining stomach. Obes Surg. 2009;19(5):664–6. 10.1007/s11695-009-9819-5. 19291339 10.1007/s11695-009-9819-5

[3] Kersebaum JN, Schafmayer C, Ahrens M, et al. Duodenal intussusception of the remnant stomach after biliopancreatic diversion: A case report. BMC Surg. 2018;18(1):57. 10.1186/s12893-018-0392-5. 30107839 10.1186/s12893-018-0392-5 PMC6092866

[4] March B, Whiting S, Karihaloo C. Gastroduodenal intussusception of remnant stomach after gastric bypass: A case report. Obes Surg. 2019;29(12):4057–9. 10.1007/s11695-019-04187-7. 31595431 10.1007/s11695-019-04187-7

[5] Freitas D, Saunders J, Parikh M. Gastrogastric and gastroduodenal intussusception after gastric plication. Obes Surg. 2024;34(10):3924–5. 10.1007/s11695-024-07499-5. 39245698 10.1007/s11695-024-07499-5

